# Health workers’ outreach and intention to use contraceptives among married women in India

**DOI:** 10.1186/s12889-020-09061-1

**Published:** 2020-06-30

**Authors:** Abhishek Kumar, Anrudh K. Jain, Faujdar Ram, Rajib Acharya, Ankita Shukla, Arupendra Mozumdar, Niranjan Saggurti

**Affiliations:** 1 India Habitat Centre, Population Council, India Office, Zone 5A, Ground Floor, Lodi Road, New Delhi, Delhi, 110003 India; 2 Former distinguished Scholar, Population Council, New York, USA; 3 Former Director, International Institute for Population Sciences, Mumbai, India

**Keywords:** Health workers, Outreach for family planning, Intention to use contraceptive, Pooled multivariate analysis, India

## Abstract

**Background:**

The influence of health workers on uptake of maternal healthcare services is well documented; however, their outreach for family planning (FP) services and influence on the intention to use contraceptives is less explored in the Indian context. This study examined the extent of health worker outreach for FP service and its effects on intention to use contraceptives among currently married women aged 15**–**49 years.

**Methods:**

This study used data from two rounds of the National Family Health Survey (NFHS) of India, conducted during 2005**–**06 and 2015**–**16 respectively. Bivariate analysis and multivariate logistic regression were used to understand the level of and change in health worker outreach for FP services over time, and its association with intention to use contraceptives among currently married women.

**Results:**

In the past 10 years, health workers’ outreach for FP service has significantly increased by about 10 percentage points, although the level is not optimal and only 28% of non-users were reached by health workers in 2015**–**16. Increase in the outreach to younger and low parity women was higher than their respective counterparts. Intention to use contraceptive among women who were not using any method was 41% when health workers contacted and discussed FP, compared to only 20% when there was no such contact with health workers. Multivariable analysis suggests that contact with health workers has significant positive effects on intention to use contraceptive (AOR = 3.05; *p* < 0.001; 95% CI 2.85–3.27).

**Conclusion:**

Increased scope of outreach of frontline health workers to provide FP communication and services will not only help in building knowledge of contraceptive methods but will also increase women’s intention to use a method. For India, this may be the most promising way to achieve the Sustainable Development Goals 3.7, which calls for universal access to reproductive health services.

## Background

For the past several decades, community health workers (CHWs) have provided primary healthcare services across the globe, including reproductive, maternal, newborn and child healthcare (RMNCH) services and their efforts have helped in reducing maternal and child mortality [1]. The CHWs have also been instrumental in reducing unmet need for contraception in many developing countries [2, 3]. While studies have supported the role of CHWs in increased uptake of RMNCH services, their contact with clients for family planning (FP) has been limited, especially in India [4–6] and the studies examining the role of health worker outreach in women’s intention to use contraceptive has been hitherto unexplored.

In India, CHWs are mostly comprised of Auxiliary Nurse Midwives (ANMs), Accredited Social Health Activists (ASHAs) and Anganwadi workers. ANMs—1 ANM for every 5000 population— are primarily located in health sub-centers; whereas, ASHAs and Anganwadi workers—1 for every 1000 population—are community-based health workers from the local community who are the first points of contact for rural women to receive information on healthcare in villages. The role of CHWs in increasing the utilization of maternal, newborn and child health services has been widely acknowledged [4–8].

In India, the CHWs have been continuously involved in ongoing reproductive and child healthcare programs over the last few decades, including FP; though little is known about the extent of their outreach in providing FP messages and services. Furthermore, there is limited evidence whether or not the CHWs outreach increases demand for contraception in the community. Therefore, the present study intended to examine the extent of CHWs FP outreach and its influence on demand for contraception among married women in India. The study also examined the temporal change in the outreach as it will reflect the progress made over time. It also anticipates that the evidence generated will help in guiding effective CHWs engagement in the ongoing FP programs in India. This will be particularly true for the Mission Parivar Vikas program that aims to increase contraceptive use in high fertility regions of the country and widely uses CHWs engagement for providing FP messaging and services, specifically among newly married women and couples [9].

## Methods

### Data

Data used in this study are from the third and fourth round of the National Family Health Survey (NFHS) conducted in India during 2005**–**06 and 2015**–**16. For convenience, we refer NFHS 2005**–**06 as NFHS 3; and NFHS 2015**–**16 as NFHS 4. The NFHS is similar to the Demographic and Health Surveys (DHS) conducted in other countries. The NFHS is conducted on representative samples of households spanning the states and union territories of India, which covers more than 99% of the country’s population. The NFHS aims to provide reliable information on fertility, use of reproductive healthcare services, childhood mortality, health and nutritional status of mother and their newborns, knowledge and prevalence of HIV etc. All indicators can be estimated at national and state levels. Additionally, the NFHS 4 also provided estimates on some of the indicators at the district level.

Both rounds of the NFHS adopted a multistage sampling design – a two-stage sampling design in most of its rural areas and a three-stage design in most of its urban areas. In the NFHS 3, the information was collected from a nationally representative sample of 109,041 households and 124,385 women aged 15–49 years. Whereas, in the NFHS 4, the information was collected from 601,509 households and 699,686 women aged 15–49 years. In both rounds of the survey, data was collected using two interview schedules: household schedule and eligible women/individual schedule. The contents of the interview schedule have remained similar across the survey periods. The household response rate was 98% in each of the survey periods and the individual (women) response rate was 94% in the NFHS 3 and 97% in the NFHS 4. Details of the sampling design and sample size estimation are given elsewhere [10, 11].

### Measures

#### Dependent variable

Intention to use contraceptive (in next 12 months from the date of interview) was considered the key outcome variable. In both rounds of the survey, all currently married women who were not using any contraceptive method were asked “Do you think you will use a contraceptive method to delay or avoid pregnancy in the next 12 months?”. Those women who responded ‘yes’ to this question were considered as ‘having intention to use contraceptive’, and others as ‘no intention to use contraceptive’.

#### Key independent variable

Health workers’ outreach to women for providing FP information was considered as the key independent variable in the study. This study captured health workers outreach for FP using the following three variables:

- *Health workers ever discussed about any family planning*: This indicator is defined based on a question asked in the survey – “Were you ever told by a health worker about any methods of family planning that you can use to avoid pregnancy?” This question is posed to women aged 15–49 years who never used any FP services at the time of the survey.
- *Health workers contacted in past three months*: This indicator is measured by combining two questions asked in the survey. The first question is “In the last three months have you met with an Auxiliary Nurse Midwives or Lady Health Visitors?”. Those women who responded ‘no’ to this question were further asked “In the last three months, have you met with an Anganwadi worker, Accredited Social Health Activist or other community health worker?” These two questions were posed to all women aged 15–49 years. Those women who responded ‘yes’ to either of the questions were considered as contacted by health workers in last 3 months prior to the survey date. Unlike the previous indicator, this indicator captured the most recent contact with health workers for any purpose and not specifically for discussing family planning.
- *Health workers contacted in past 3 months and ever discussed family planning:* This indicator is computed based on the two aforesaid indicators. Those women who had been in contact with health workers in the past 3 months and the health worker had discussed FP during the contact are considered under this indicator.

These three variables together reflect anytime outreach, current outreach, and health worker’s potential missed opportunity for discussing FP during most recent contact with a woman.

#### Confounding variables

To assess the association between health workers’ outreach to women and women’s intention to use contraceptives, the following variables were controlled in multivariate analyses: age of the women (15–24 years, 25–34 years, 35–49 years), number of living children/parity (0 child, 1 child, 2 children, 3+ children), women’s education (uneducated, 1–10 years of schooling, 10+ years of schooling), current working status (no, yes), place of residence (rural, urban), household wealth quintiles (poorest, poorer, middle, richer, richest), caste (Scheduled Caste [SC], Scheduled Tribes [ST], Other Backward Caste [OBC], Others), religion (Hindu, Muslims, Others), and region/state of the country. Regions of the country were included to adjust for geographical variations in the outcome variable. All these variables were considered in the current study given the evidence of their association with contraceptive use from previously published studies [12–14].

### Statistical analysis

Univariate analysis was used to understand the extent of health workers’ outreach for FP over time. Bivariate analysis was used to understand the socioeconomic profile of the women and also to understand the disparity in health workers’ outreach across selected sociodemographic characteristics of women. Furthermore, it was also useful to understand the association of health workers’ outreach with intention to use contraceptives.

To understand the effect of health workers’ outreach on intention to use contraceptives, the study used multivariate logistic regression with data from both rounds of the survey. It estimated the interaction effect of the survey time period and health workers’ contact on the outcome variable. This was done to understand the temporal as well as net effect of health workers’ outreach on intention to use contraceptives. Four categories for the interaction term were used: (1) year 2005**–**06 and no contact (reference category), (2) year 2005**–**06 and contact, (3) year 2015**–**16 and no contact, and (4) year 2015**–**16 and contact; where the second category reflected the net effect of contact in 2005–06, the third category reflected the temporal change in the absence of any contact, and the fourth category reflected the effect of contact after taking into account the temporal change. The regression analyses were adjusted for the selected sociodemographic variables. Results obtained from the regression analysis were presented in terms of odds ratios (OR) and adjusted odds ratios (AORs), with 95% confidence intervals (CI) and corresponding significance level. The AOR can be interpreted as, for instance, AOR > 1 indicates higher odds of intention to use contraceptives while AOR < 1 indicates lower odds of intention to use contraceptives. The analysis presented hereafter were carried using analytical software STATA 13.0. All the analyses were conducted on currently married women 15–49 years who were not using any contraceptive at the time of survey in both the survey rounds. The number of such women – analytical sample size for this study – were 37,296 in the NFHS 3 and 247,024 in the NFHS 4.

## Results

### Socioeconomic characteristics of the study sample

Distribution of the respondents—currently married women who were not using any family planning services—by age group has changed over the survey periods. For instance, proportion of the women aged 15–24 years reduced from 42% in 2005–06 to 31% in 2015–16; however, the proportion across older age groups increased (Table 1). Proportion of uneducated women decreased from 52 to 33%, while proportion of women who received 10+ years of schooling increased from 10 to 22% during 2005**–**06 and 2015**–**16 respectively. Over the survey periods, minimal change was noticed in distribution of women by household wealth – the proportion decreased, though slightly, across poor households whereas increased across rich households. The sample distribution by geographic regions of the country were more or less similar over the survey periods.

**Table 1:** Percentage distribution of currently married women (15–49 years) who are not using any family planning method by selected demographic and socioeconomic characteristics, India, 2005**–**16

Characteristics	2005–06	2015–16	*P*-value*
**Age of women**			
15–24 years	41.9	30.7	< 0.001
25–34 years	31.9	36.9	< 0.001
35–49 years	26.2	32.4	< 0.001
**Parity**			
0 child	23.1	20.7	< 0.001
1 child	25.0	27.1	0.899
2 children	19.0	24.6	< 0.001
3 + children	32.9	27.7	< 0.001
**Education**			
Uneducated	52.0	32.8	< 0.001
1–10 years of schooling	38.6	45.7	< 0.001
10+ years of schooling	9.5	21.5	< 0.001
**Current working status**			
Not working	60.6	76.2	< 0.001
Working	39.4	23.8	< 0.001
**Place of residence**			
Rural	74.6	69.2	< 0.001
Urban	25.4	30.8	< 0.001
**Household wealth quintiles**			
Poorest	24.8	22.6	< 0.001
Poorer	22.3	20.4	< 0.001
Middle	19.9	19.5	0.465
Richer	17.5	19.4	< 0.001
Richest	15.6	18.1	< 0.001
**Caste**			
Scheduled Castes	19.7	20.6	< 0.05
Scheduled Tribes	10.0	10.4	< 0.001
Other Backward Castes	43.1	47.8	< 0.001
Other	27.1	21.2	< 0.001
**Religion**			
Hindu	78.8	79.9	< 0.001
Muslim	16.4	15.5	< 0.001
Other	4.9	4.6	< 0.001
**Region of the country**			
North	13.2	11.0	< 0.001
Central	28.2	25.2	< 0.001
East	26.4	25.9	< 0.001
Northeast	3.7	3.7	< 0.001
West	11.1	12.7	< 0.001
South	17.5	21.5	< 0.001
**Total number (unweighted)**	**37,296**	**247,024**	

^*^P-values are obtained by applying proportion test for comparing percentage distribution of women across survey rounds by sub-groups of each of the characteristic

### Health workers outreach for family planning

**Fig. 1:**
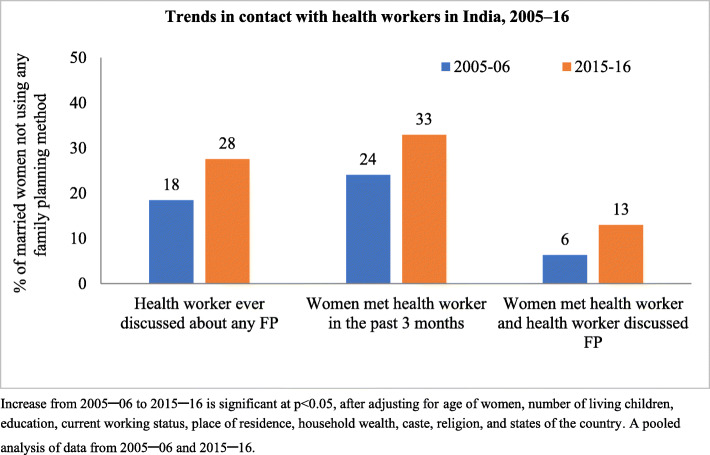
Trends in health worker outreach among currently married women (15–49 years) who were not using any family planning method at the time of survey, India, 2005–16

Women who reported ever discussing FP with a health worker increased from 18% in 2005**–**06 to 28% in 2015**–**16 (Fig. 1). A similar increase was observed in other outreach indicators. In 2015**–**16, 33% of non-user women were contacted by the health workers in the past 3 months, however only in 13% of cases there was discussion on FP during the contact.

The extent of health worker outreach varied by age, parity, education and household economic status of the women, place of residence and religion (Table 2). In 2015**–**16, the extent of health workers ever discussed FP among women without schooling (21%) was lower than among women with 10+ years of schooling (33%). It was 21% among women of poorest households as compared to 32% among the richest households. While looking at temporal increase in the health worker outreach across the subgroups of women, the increase was highest among younger and low parity women. For instance, the extent of ever discussion on FP increased by 13 percentage points among women aged 15–24 years as compared to only by 2 percentage points among women aged 35–39 years. Similarly, the contact increased by 13 percentage points among women with 0 child, 14 percentage points among women with 1 child and only by 1 percentage point among women with 3 and more children. The pattern was similar for other indicators of the outreach.

**Table 2:** Health worker outreach among currently married women (15–49 years) who were not using any family planning methods by selected demographic and socioeconomic characteristics, India, 2005**–**16

Survey year	Percentage of women ever discussed FP with health workers			Percentage of women who had contact with health worker in past 3 months			Percentage of women who had contact in past 3 months and discussed FP with health worker		
	2005–06	2015–16	Differences during (2016–2005)	2005–06	2015–16	Differences during (2016–2005)	2005–06	2015–16	Differences during (2016–2005)
**Age of women**									
15–24 years	13.7	27.0	13.3	27.9	44.1	16.2	6.4	16.3	9.8
25–34 years	22.0	31.0	9.0	28.9	37.6	8.7	8.5	15.9	7.4
35–49 years	21.7	24.1	2.4	12.0	16.9	5.0	3.4	6.5	3.1
**Parity**									
0 child	8.0	20.8	12.8	12.2	25.6	13.4	1.8	8.3	6.6
1 child	18.4	32.4	14.0	28.9	42.5	13.7	7.2	17.4	10.3
2 children	23.4	31.9	8.5	29.3	34.5	5.2	9.0	15.1	6.1
3 + children	22.8	24.0	1.1	25.6	27.6	2.0	7.2	10.1	2.9
**Education**									
No schooling	14.7	20.5	5.8	23.8	27.7	3.9	5.0	8.8	3.8
1–10 years of schooling	21.4	30.1	8.7	25.6	36.0	10.4	7.8	14.9	7.1
10+ years of schooling	26.3	32.8	6.5	18.8	34.2	15.4	7.5	15.2	7.8
**Currently working**									
No	17.8	27.9	10.1	23.3	34.0	10.7	5.9	13.5	7.6
Yes	19.4	29.5	10.1	25.1	32.0	6.9	6.9	13.8	6.9
**Place of residence**									
Rural	17.1	26.0	9.0	27.3	35.8	8.5	6.9	13.5	6.5
Urban	22.3	30.9	8.6	14.3	26.4	12.1	4.4	11.8	7.4
**Wealth quintile**									
Poorest	14.7	20.9	6.2	27.2	34.5	7.3	5.8	10.9	5.1
Poor	17.5	26.1	8.6	27.4	35.6	8.3	7.1	13.5	6.4
Middle	18.1	29.1	11.0	25.2	35.0	9.8	7.1	14.3	7.2
Rich	19.6	30.9	11.3	23.0	32.4	9.4	6.3	14.2	7.9
Richest	24.6	32.2	7.5	13.8	26.1	12.3	5.0	12.2	7.2
**Caste**									
Scheduled Castes	18.3	28.0	9.7	27.8	36.4	8.6	7.4	14.2	6.9
Scheduled Tribes	17.4	28.1	10.7	26.3	36.2	9.9	7.3	15.1	7.8
Other Backward Caste	17.6	27.1	9.5	23.9	32.7	8.8	5.9	12.6	6.7
Other	20.1	28.5	8.3	20.7	28.6	8.0	5.6	11.6	6.0
**Religion**									
Hindu	18.5	27.7	9.2	24.3	32.8	8.5	6.4	13.0	6.6
Muslims	17.2	24.7	7.5	24.0	33.4	9.5	5.7	11.6	6.0
Other	21.4	33.7	12.4	20.4	33.6	13.2	6.7	16.1	9.4

*Differences during 2016–2005 > =5% is significant at p < 0.05*

### Health workers’ outreach and intention to use contraceptives

Intention to use contraceptives was found to be significantly higher among those women who were contacted by health workers than those who were not (Table 3). In 2015**–**16, the intention to use contraceptive was 31% among those women who were reached by health workers and took part in FP discussions, as compared to 20% among those who were not. Similarly, the intention to use contraceptives was 41% when women were contacted in the past 3 months and ever discussed about any FP compared to only 20% in otherwise situation.

**Table 3:** Differences (%) in intention to use contraceptive^ by health worker outreach among currently married women (15–49 years) not using any family planning method, India, 2005**–**16

Survey year	2005–06	2015–16
**Health worker ever discussed about any FP**		
No	16.7	19.5
Yes	25.6	31.1
**Health worker contacted in past 3 months**		
No	15.7	15.9
Yes	26.8	36.4
**Health worker contacted in past 3 months & ever told about FP**		
No	17.3	19.9
Yes	34.4	41.4
**Total intention to use contraception in next 12 months**	**18.4**	**22.8**

^ ref. period – 12 months from the date of interview

### Effect of health workers’ outreach on intention to use contraceptives

Logistic regression analysis of pooled data from both rounds of the survey was conducted to examine the effect of health workers’ outreach on intention to use contraceptive. In comparison to women who never discussed FP with a health worker and who were interviewed in 2005**–**06, the odds of the intention to use contraceptives was higher among women who discussed FP with a health worker (AOR = 1.46; *p* < 0.001; 95% CI: 1.37–1.57), and would have increased by 32% over time among women who never discussed FP (AOR = 1.32; *p* < 0.001; 95% CI: 1.25–1.39) (Table 4). The interaction term of health workers’ contact in 2015**–**16 strongly effected intention to use contraceptives. For instance, the adjusted odds ratio of intending to use contraceptive was 2.35 (*p* < 0.001; 95% CI: 2.22–2.49) times higher among women who were reached by health workers in 2015**–**16 as compared to women who were not reached by health workers in 2005**–**06. Similarly, the adjusted odds ratio of intention to use contraceptive was 2.61 (*p* < 0.001; 95% CI: 2.47–2.76) when there was contact with health workers in the past 3 months during 2015**–**16. Furthermore, the adjusted odds ratio of intention to use contraceptive was 3.05 (*p* < 0.001; 95% CI: 2.85–3.27) when FP was discussed during contact with health worker in the past 3 months during 2015**–**16.

**Table 4:** Logistic regression analysis showing the interaction effect of survey period and contact with health worker outreach on intention to use contraceptive^ among currently married women (15–49 years) not using any family planning method, India, 2005**–**16

	% of non-user intended to use contraceptives	Unadjusted odds ratio of intention to use contraceptives	Adjusted# odds ratio of intention to use contraceptives
**Interaction between survey period and ever discussed about any FP**			
2005**–**06 × no contact	16.7	Ref.	Ref.
2005**–**06 × contact	25.6	1.56 (1.46, 1.65)***	1.46 (1.37, 1.57)***
2015**–**16 × no contact	19.5	1.18 (1.15, 1.22)***	1.32 (1.25, 1.39)***
2015**–**16 × contact	31.1	2.26 (2.19, 2.34)***	2.35 (2.22, 2.49)***
**Interaction between survey period and contact in past 3 months**			
2005**–**06 × no contact	15.7	Ref.	Ref.
2005**–**06 × contact	26.8	1.91 (1.79, 2.02)***	1.33 (1.24, 1.42)***
2015**–**16 × no contact	15.9	0.97 (0.94, 1.01)	1.09 (1.04, 1.15)**
2015**–**16 × contact	36.5	3.08 (2.98, 3.19)***	2.61 (2.47, 2.76)***
**Interaction between survey period and contact in past 3 months ever told FP**			
2005**–**06 × no contact	17.3	Ref.	Ref.
2005**–**06 × contact	34.4	2.29 (2.08, 2.52)***	1.67 (1.51, 1.86)***
2015**–**16 × no contact	19.9	1.17 (1.14, 1.21)***	1.32 (1.26, 1.38)***
2015**–**16 × contact	41.4	3.48 (3.35, 3.60)***	3.05 (2.85, 3.27)***

^ ref. period – 12 months from the date of interview

Ref. reference category

**p < 0.05; ***p < 0.001

Figures in parenthesis are 95% confidence intervals

#adjusted for age of women, parity, education, current working status, place of residence, household wealth quintile, caste, religion and state of the country

## Discussion

Using two latest rounds of the National Family Health Survey (NFHS) conducted between 2005 and 2016, this paper examined change in the community health workers’ FP outreach among currently married women who were not using any FP services at the time when the survey(s) were conducted. Furthermore, it examined the influence of outreach on intention to use contraceptives. The findings show that health workers’ FP outreach increased over the span of 10 years between the NFHS 3 and 4. However, it must be noted that the current level of outreach was found to be far from universal. Moreover, even if contact was made, discussion on FP during the contact was very low. The findings further indicated that health workers’ FP outreach had a positive and significant effect on intention to use contraceptives.

The findings of this study are similar to that of previous studies conducted in India which documented that the contact and advice on FP between the providers and clients was very low compared to advice on antenatal and delivery care [15–17], indicating low FP outreach by health workers. This could be possible due to intensive focus on maternal and child healthcare services rather than FP by the Indian health programs in the past two decades. For instance, after 2000, India launched two ambitious health programs under the flagship programs Janani Suraksha Yojana and National Rural Health Mission of the Ministry of Health and Family Welfare, Government of India. These programs aimed at curbing the vulnerabilities of maternal, neonatal and infant mortality through increased institutional delivery, antenatal care and postnatal checkups [18]. Thus, emphasis on improving the maternal and child health (MCH) services might have affected the CHWs’ involvement in family planning. Another possible explanation for the low outreach and discussions on FP might be associated with lower monetary incentives for FP, especially for ASHA workers, than for MCH related work. With the exception of female sterilization, CHWs, particularly ASHAs, received lower activity based monetary remunerations and incentives for FP outreach as compared to antenatal, delivery, and post-natal healthcare work.

An encouraging aspect of the program was the significant increase in the health workers’ FP outreach to low parity and young women than older women in the last ten years. Findings further showed that contact for FP was higher among educated and richest than uneducated and poorest women. Health workers’ FP outreach did not vary based on social status like caste or religion of women and the pattern was similar over time. This finding was in line with the results of a previous study conducted in India [19].

The findings clearly showed that health workers’ outreach had a significantly positive effect on the intention to use contraceptives among women who were not using any FP services at the time of survey. While the type of information exchanged between the health workers and women during the contact is not known from the data set, it can be assumed that contact with health workers may have increased knowledge among non-users about different contraceptive methods and places where FP services were available. Furthermore, during the contact, health workers might have generated demand for FP among the non-users by offering counselling on side effects and removing myths and misconceptions about contraceptive methods, which is one of the reasons for not using FP services among women in developing countries [20, 21].

Although findings offer important insights, they need to be interpreted cautiously considering few limitations. First, the study used cross-sectional survey data, which can only reveal association rather than causal effect between outcomes and covariates. Second, this study did not account for the quality of community health workers’ outreach or matters discussed during the contact, as this information was not available in the cross-sectional NFHS data. Third, both rounds of the surveys are cross-sectional and samples from each round of the survey were selected independently; therefore, there was only a minimal chance of the same women being surveyed in both rounds. Finally, there may be important unmeasured factors that explain the observed associations, that were not captured in the dataset used in this study.

## Conclusion

The findings of this study offer important policy and research implications in context of the ongoing family planning programs and initiatives in India. First, there is a need to increase involvement of community health workers for FP counselling, demand generation, and service provision in the community. This could be achieved by providing additional training to health workers, emphasizing importance of contraceptive use, motivating and encouraging them to conduct FP related work. Furthermore, this could also be attained through a system of incentives to change the behavior of health workers to include required education (particularly of ASHA) and advocacy to increase use of modern contraceptives. Second, health workers should be encouraged to reach young and low parity, uneducated, and poor women and generate demand for FP among them through counselling and providing contraceptives. This will result in an average increase in contraceptive use in the country and help achieve the universal access to reproductive health services by 2030 – the Sustainable Development Goals 3.7. Third, information on health workers’ FP outreach should be collected from both users and non-users of contraceptive methods to generate further evidence on the effect of health workers’ FP outreach on contraceptive use.

## Data Availability

Data would be made available by the corresponding author(s) upon reasonable request.

## Abbreviations

AOR: Adjusted Odds Ratio
ANM: Auxiliary Nurse Midwives
ASHA: Accredited Social Health Activist
CHW: Community Health Worker
CI: Confidence Intervals
FP: Family Planning
MCH: Maternal and Child Health
NFHS: National Family Health Survey
RMNCH: Reproductive, Maternal, Newborn, Child Health
